# Enhancing tumor control in liver metastases treated with SBRT: dosimetric predictors and clinical outcomes from a single-center analysis

**DOI:** 10.1007/s10585-025-10344-3

**Published:** 2025-04-26

**Authors:** Lisa Seyfried, Michael J. Eble, Ahmed Allam Mohamed

**Affiliations:** 1 Department of Radiation Oncology, Medical Faculty, RWTH Aachen University, Aachen, Germany; 2 Center for Integrated Oncology Aachen, Bonn, Cologne and Duesseldorf (CIO ABCD), Aachen, Germany; 3 Universitätsklinik RWTH Aachen, Pauwelstraße 30, 52074 Aachen, Germany

**Keywords:** Stereotactic ablative body radiotherapy (SABR), Metastasis directed therapy, Tumor control probability, Tumor response modeling, Liver tumors

## Abstract

Liver metastases, a hallmark of systemic disease, carry a poor prognosis despite advancements in systemic therapies. Stereotactic body radiation therapy (SBRT) has emerged as a promising local treatment, offering durable tumor control with minimal toxicity. However, the optimal dosimetric strategies to maximize outcomes remain an area of active investigation. This retrospective study evaluated 76 patients with 101 liver metastases treated with SBRT between November 2012 and June 2024. Dosimetric parameters were analyzed, including prescribed dose (PD) and dose metrics for planning target volume (PTV) and gross tumor volume (GTV), with doses converted to equivalent doses in 2 Gy fractions (EQD2, α/β = 10). Tumor control probability (TCP) models and survival outcomes were assessed, with a focus on the prognostic impact of dosimetric and clinical factors. Median overall survival (OS) was 33 months, with 1-year and 3-year OS rates of 74.1% and 39.4%, respectively. Freedom from local progression (FFLP) was 82.5% at 12 months. PD emerged as the strongest independent predictor of local control, with an optimal threshold of 77.44 Gy EQD2 significantly improving 1-year FFLP rates (96.8% vs. 67.2%; p = 0.007). Advanced motion management techniques, including internal breath-hold (iBH) with image-guided radiotherapy (IGRT), demonstrated superior local control outcomes. Predictive modeling confirmed PD as the most robust dosimetric metric, correlating with a high TCP and outperforming other dose metrics. Toxicity was minimal, with only 3.9% experiencing grade ≥ 3 adverse events. SBRT represents a highly effective and safe approach for liver metastases, with PD and advanced imaging emerging as pivotal determinants of tumor control. These findings underscore the importance of precise dosimetric planning and motion management in optimizing SBRT outcomes. This study provides a robust framework for personalized treatment strategies, contributing to the integration of SBRT as a cornerstone in the multidisciplinary management of liver metastases.

**Supplementary Information:**

The online version contains supplementary material available at 10.1007/s10585-025-10344-3.

## Introduction

Liver metastases are detected synchronously in approximately 5% of cancer patients [1]. Among younger individuals, colorectal and breast cancers are the most common primary sites, while in older patients, liver metastases originate from a broader spectrum of cancers, including esophageal, stomach, and bladder cancers [1, 2]. Prognostically, liver metastases are associated with significantly poorer outcomes, with a 1-year survival rate of only 15.1% compared to 24.0% in patients with non-hepatic metastases [1]. This poor prognosis is partly attributed to the liver's unique biology, characterized by its hemodynamic architecture. It includes slow and tortuous microcirculation and liver sinusoidal endothelial cells that promote tumor cell attachment and retention. Furthermore, the liver's regenerative capacity and regionally immunosuppressive microenvironment create a favorable niche for tumor cell survival, growth, and metastasis development [3].

Systemic therapies are typically the first-line approach for managing liver metastases; however, local therapies become increasingly critical during the disease course, particularly in cases of oligometastases, or to provide patients with a chemotherapy-free interval [4, 5]. Surgery has long been considered the gold standard for treating liver metastases, but many patients are ineligible due to contraindications. In such scenarios, local treatment options, including SBRT, have gained prominence as highly effective alternatives. SBRT, in particular, has emerged as a pivotal treatment modality due to its precision, efficacy, and non-invasive nature [4–6].

SBRT has emerged as an effective and increasingly utilized treatment modality for liver metastases, supported by a wealth of retrospective and prospective studies. Numerous Phase I and II trials have demonstrated variable but promising efficacy of SBRT in achieving local tumor control, with rates ranging from 5 [7–15], depending on tumor size, dose regimen, and patient selection criteria [7–10, 12, 15]. Technological advancements, particularly the incorporation of image-guided radiation therapy (IGRT) and the advent of magnetic resonance-guided linear accelerators (MR-Linacs), have further refined the precision and feasibility of delivering high-dose radiation to focal liver targets [16–21]. These innovations have allowed for improved tumor visualization, real-time motion management, and enhanced sparing of adjacent critical structures, thereby reducing treatment-related toxicities and expanding the applicability of SBRT in challenging cases [21, 22].

In the current study, we aimed to evaluate the efficacy and safety of SBRT in the local control of liver metastases, leveraging our single-institution experience. This study was planned to validate the role of SBRT as a potent local therapy for achieving high tumor control rates while maintaining a favorable toxicity profile. Furthermore, we aimed to develop predictive models for tumor control by analyzing the relationship between radiation dose and treatment outcomes. By addressing these objectives, this study seeks to provide insights into the optimization of SBRT protocols and identify key factors influencing treatment efficacy, contributing to the advancement of personalized therapeutic strategies for liver metastases.

## Materials and methods

Following approval from the local ethics committee (XX University, Faculty of Medicine, EK 23-264), a retrospective analysis was conducted involving patients with liver metastases who underwent SBRT as part of their treatment plan between November 2012 and June 2024. All cases were reviewed in a multidisciplinary tumor board, and SBRT or thermal ablation was considered for patients who were not candidates for surgical resection but were eligible for localized therapy. The decision to utilize SBRT was influenced by factors including the tumor's anatomical location (e.g., proximity to the liver dome or major blood vessels), lesion size, and contraindications to anesthesia.

This study included patients who received liver-targeted SBRT in accordance with the German Society for Radiation Oncology guidelines [23]. Exclusion criteria included (1) SBRT delivered solely for palliative purposes and (2) a lack of survival data.

### SBRT planning and delivery

Patients were evaluated for their ability to perform an inspiratory breath-hold (iBH) as part of the simulation process. Those capable of maintaining iBH for 20–30 s were treated in iBH mode, while others underwent 4D CT simulation and treatment. Planning CT (P-CT) scans were conducted using a 16-slice CT scanner (Brilliance CT Big Bore Oncology, Philips Medical Systems, Cleveland, Ohio, USA) with patients positioned on vacuum cushions for stability. To enhance breathing regularity during both simulation and treatment, patients utilized goggles connected to an optical surface scanning system (CRAD, Uppsala, Sweden).

Contrast-enhanced CT imaging in arterial and venous phases was acquired in alignment with the respiratory phase used for the P-CT. When required, fiducial markers were inserted under CT or ultrasound guidance at least one week prior to P-CT acquisition. Diagnostic and planning images were imported into the treatment planning system (Pinnacle, V.14.0, Philips Healthcare, Amsterdam, Netherlands). For patients treated with 4D CT, the internal target volume (ITV) was derived by combining the GTV across respiratory phases (0%, 50%, 90%). The planning target volume (PTV) was defined by adding a 5-mm isotropic margin to the ITV, or clinical target volume (CTV).

Radiation doses were prescribed to the 83–67% isodose line, ensuring dose heterogeneity within the PTV while prioritizing target coverage and adhering to organ-at-risk constraints (Supplementary Table 1) [24]. Radiation doses of 37.5 to 66 Gy were offered in 3, 5, 8, or 12 fraction schedules based on tumor size and location (Supplementary Table 2). SBRT was delivered using flattening filter-free (FFF) volumetric modulated arc therapy (VMAT) in 3–4 sessions per week. When organ-at-risk (OAR) constraints could not be met (in 11 lesions), the prescribed dose was adjusted to ensure compliance with these constraints based on the preference of the treating physician. Treatment was conducted with image-guided radiation therapy (IGRT) using cone-beam CT (CBCT) (XVI, Elekta, Stockholm, Sweden), performed prior to each fraction either as iBH or free breathing (FB) based on P-CT.

**Fig. 1:**
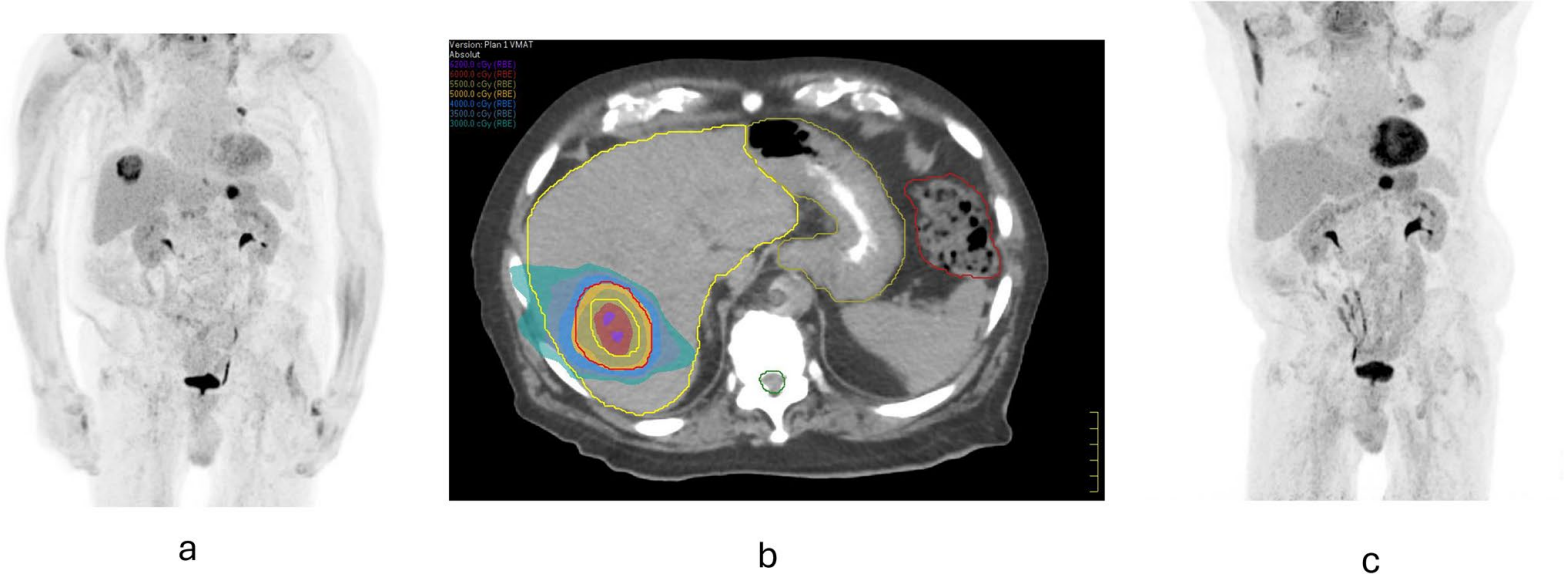
**a** Pre-treatment PET-CT demonstrating hypermetabolic activity in a liver metastasis segment. **b** Axial planning CT scan with radiation treatment plan showing the dose distribution over the target lesion in the liver. color wash lines represent different isodose levels, with adequate sparing of adjacent organs at risk (OARs). **c** Three months Post-treatment PET-CT showing the complete metabolic response of the lesion following SBRT, with reduced hypermetabolic activity

One month after SBRT, patients underwent clinical and serological evaluations, including a complete blood count (CBC), liver function tests (LFTs), and tumor markers. Imaging assessments were performed three months post-SBRT (Fig. 1) and subsequently every three months or adjusted based on individual case scenarios. Toxicity was assessed according to the National Cancer Institute's Common Terminology Criteria for Adverse Events (CTCAE) V5.

### Statistical analysis

The primary endpoint of the analysis was freedom from local progression (FFLP), evaluated at the lesion level as the time from the initiation of radiation therapy to either local progression or censoring. Overall survival (OS) was defined as the duration from the start of SBRT to death or the date of last follow-up. To identify statistically significant thresholds, receiver operating characteristic (ROC) curve analysis was performed. Kaplan–Meier methods were utilized to estimate survival outcomes, while univariate and multivariate analyses were conducted using the Cox proportional hazards model to calculate hazard ratios (HRs) and 95% confidence intervals (CIs). Additionally, tumor control probability (TCP) within one year was modeled using logistic regression to evaluate FFLP probabilities as a function of different radiation parameters to PTV. Given the variability in fractionation among patients, radiation doses were converted to equivalent doses in 2-Gy fractions (EQD2) using an α/β ratio of 10 Gy. A total of 5000 bootstrap resamples were employed to generate 95% confidence intervals for model validation. For each model, the estimated coefficient, standard error, p-value, residual deviance, and Akaike Information Criterion (AIC) were calculated to assess the significance and goodness-of-fit. Statistically significant predictors of outcomes were identified using a p-value threshold of < 0.05.Model performance metrics, including residual deviance and AIC, indicated that the models adequately fit the data, with lower AIC values reflecting better model performance.

The statistical analysis and graphics were executed using the R software version 3.4.

## Results

A total of 88 patients who underwent SBRT for liver metastases were initially identified. Of these, nine patients were excluded due to a purely palliative treatment intent, and three additional patients were excluded because survival data were unavailable, including one patient who passed away during an elective cardiac catheterization before undergoing treatment evaluation after SBRT, with his death being unrelated to radiation therapy. Consequently, the final cohort consisted of 76 patients with 101 distinct lesions, as detailed in Table 1.

**Table 1:** Summarizes the characteristics of the patient cohort, including demographic, clinical, and dosimetric parameters

Characteristics	
Number of patients	76
Number of lesions	101
Median age (range)	66 (32–93)
Gender	
Male	37
Female	39
Primary site	
Colorectal cancer	38
Pancreatobiliary cancer (including extrahepatic ccc)	13
Lung cancer	5
Breast Cancer	5
Others	15
Oligopmetastatic disease (OMD)	67
Oligoprogressive disease	9
Patients with prior / concurrent systemic therapy for the metastatic disease	
Prior	61
Concurrent	9
No	15
Type of systemic therapies	
Chemotherapies	55
Targeted therapies (including Ab anf TKI)	18
Immunotherapy	2
Hormonal treatment	2
Median physical prescribed dose (range) Gy	45 (30–66)
Median number of fractions (range)	5 (3–12)
Dose prescribed to isodose line, Median (range)	80% (67–83%)
Median prescribed dose (PD) to PTV periphery	
As EQD2 _α/ β 10_ (range) Gy	70.3 (40- 93.75)
As BED 10 Gy	84.38 (48–112.5)
Median maximal dose in PTV (PTVmax)	
As EQD2 _α/ β 10_ (range) Gy	115.96 (67.19–189.07)
As BED 10 Gy	138.88 (81.34–227)
Median near maximum dose of PTV (PTV D2%)	
As EQD2 _α/ β 10_ (range) Gy	113.42 (64.72–184.42)
As BED 10 Gy	136.45 (77.55- 221.03)
Median of PTV median dose (PTV D50%)	
As EQD2 _α/ β 10_ (range) Gy	100.84 (53.61–149.83)
As BED 10 Gy	120.02 (64.38- 179.58)
Median near minimum dose of PTV (PTV D98%)	
As EQD2 _α/ β 10_ (range) Gy	71.2 (22–93.85)
As BED 10 Gy	85.5 (27.2–102.11)
Median GTV volume (range) cm^3^	18.3 (0.7–257.8)
Median PTV volume (range) cm^3^	53.4 (7.3–456.4)
IGRT with CBCT in	
FB	51
iBH	50

*OMD* oligometastatic disease, *Ab* Antibodies, *TKI* Tyrosine kinase inhibitors EQD2, equivalent dose in 2 Gy fractions (α/β = 10), *PTV* planning target volume, *GTV* gross tumor volume, *iBH* internal breath-hold, *FB* free breathing

The median age of the patients was 66 years, with a nearly equal distribution of males (37) and females (39). The primary tumor origins included colorectal cancer (CRC) in 38 cases (50%), while the remaining cases were non-colorectal cancers (non-CRC), comprising pancreaticobiliary cancers (pancreatic cancer and extrahepatic cholangiocarcinoma) in 13 patients, breast cancer in 5 patients, lung cancer in 5 patients, and a diverse group of other malignancies, including stomach cancer, anal cancer, cervical cancer, and neuroendocrine carcinoma, accounting for 15 cases.

Of the total cohort, 67 patients were treated with SBRT for oligometastatic liver disease, while 9 patients underwent SBRT for oligoprogressive liver metastases. Prior systemic chemotherapy for metastatic disease was administered to 61 patients, whereas 15 patients had not received any systemic therapy prior to SBRT.

The SBRT treatment characteristics are described in Table 1. This included a median physically prescribed dose of 45 Gy (range: 30–66 Gy), delivered in a median of 5 fractions (range: 3–12). The median isodose line for dose prescription was 80% (range: 67–83%), and the median prescribed dose (PD) to PTV, expressed as EQD2 (α/β = 10), was 70.3 Gy (range: 37.5–93.75 Gy).

### Survival outcomes and toxicities

**Fig. 2:**
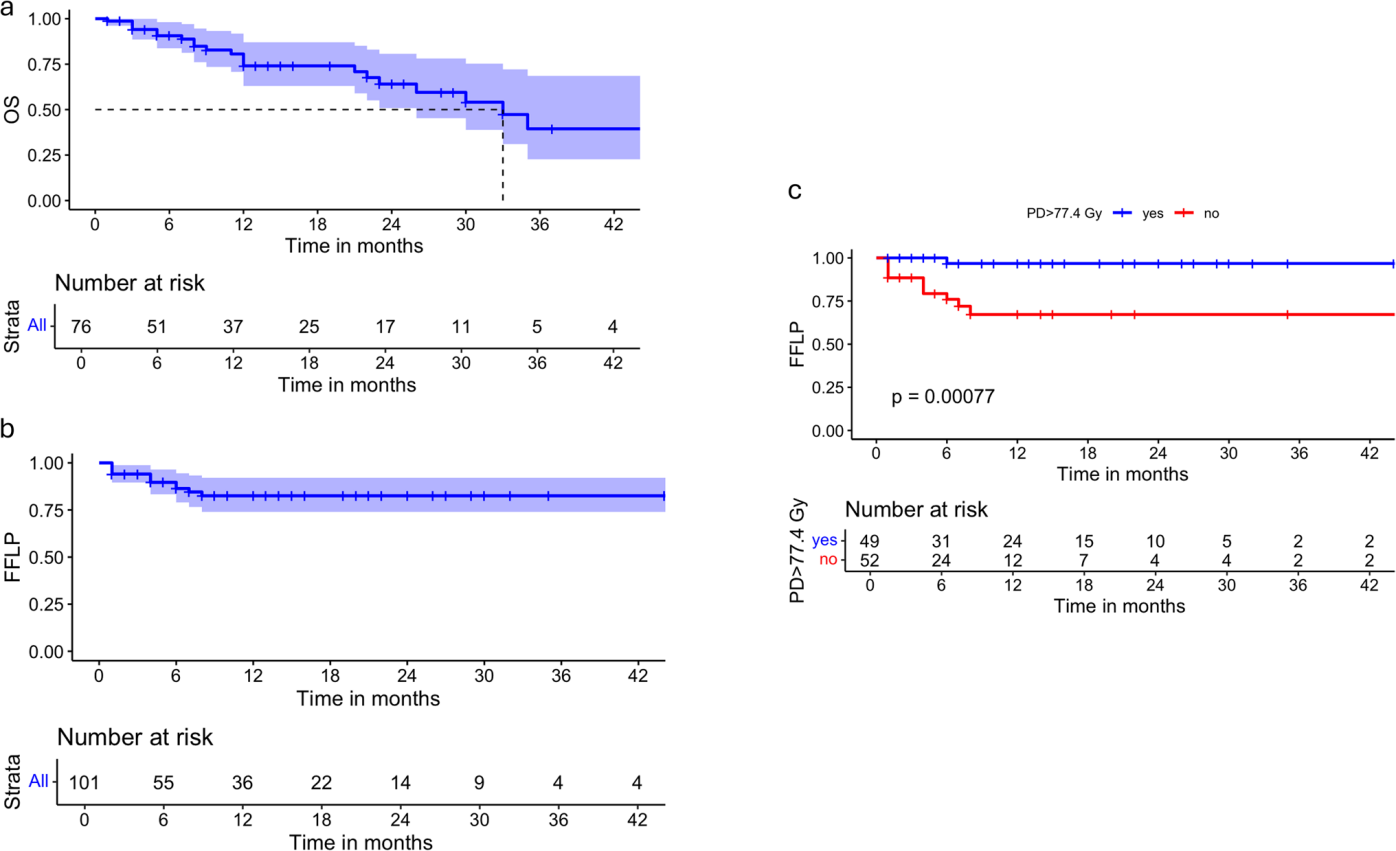
**a** Kaplan–Meier survival analyses illustrating overall survival (OS) and freedom from local progression (FFLP). **b** The OS curve for all patients, with the shaded region representing the 95% confidence interval (CI). **c** The FFLP curve for all patients, with the shaded region representing the 95% CI. **d** The FFLP curve stratified by the prescribed dose (PD > 77.4 Gy versus PD ≤ 77.4 Gy), showing a statistically significant difference in local control rates between groups (p = 0.00077, log-rank test). The number at risk for each time point is displayed below each plot

The median follow-up period was 14 months, and the median OS was 33 months (Fig. 2a). The 1-year and 3-year OS rates were 74.1% and 39.4%, respectively.

With a total of 13 local failures reported within the first 12 months, FFLP rates at 6 months was 86.4% (95% CI 79.0–94.5%) and remained stable at 82.5% (95% CI 74.0–92.1%) at both 12 and 24 months (Fig. 2b).

Cox regression analyses were performed to investigate the prognostic factors influencing FFLP. The results of the univariate analysis are summarized in Table 2. Tumor group (CRC vs non-CRC) showed no significant association with the outcome (HR 1.04, 95% CI 0.53–2.05, p = 0.918). Dosimetric parameters, including PD (HR 0.94, 95% CI 0.91–0.98, p = 0.001), PTVmax (HR 0.97, 95% CI 0.94–0.99, p = 0.012), PTV D2% (HR 0.97, 95% CI 0.94–0.99, p = 0.011), PTV D50% (HR 0.95, 95% CI 0.92–0.98, p = 0.003), PTV D95% (HR 0.95, 95% CI 0.92–0.98, p = 0.003), PTV D98% (HR 0.96, 95% CI 0.93–0.99, p = 0.007), and GTV D2% (HR 0.97, 95% CI 0.94–0.99, p = 0.015), were significantly associated with improved outcomes.

**Table 2:** Presents the results of univariate and multivariate analyses evaluating the association between various parameters and clinical outcomes

Parameter	Univariate analysis		Multivariant analysis	
	HR (95% CI)	P-value	HR (95% CI)	P-value
Primary tumor (CRC vs non-CRC)	1.04 (0.53–2.05)	0.918		
Dose per fraction	0.8 (1.18–0.68)	0.1		
PD	0.95 (0.91–0.98)	0.001	0.96 (0.92–0.99)	0.026
PTVmax	0.97 (0.94–0.99)	0.012		
PTVD2%	0.97 (0.94–0.99)	0.011		
PTV50%	0.95 (0.92–0.98)	0.003		
PTVD98%	0.96 (0.93–0.99)	0.007		
GTVD2%	0.97 (0.94–0.99)	0.015		
IGRT (FB vs iBH)	6.3 (1.39–28.55)	0.017	3.4 (0.64–16.82)	0.14
GTV in mL	1.00 (0.99–1.00)	0.922	0.997 (0.985–1.01)	0.677
PTV in mL	1.00 (0.99–1.00)	0.849		
Prior systemic therapy (yes vs no)	0.90 (0.25–3.27)	0.872		
Concurrent systemic therapy (yes vs no)	< 0.01 (< 0.01–0)	0.4		

Hazard ratios (HR) with 95% confidence intervals (CI) and corresponding p-values are shown

*CRC* colorectal cancer, *PD* prescribed dose as equivalent in 2 Gy fractions (α/β = 10), *PTVmax* maximum dose to the planning target volume in EQD2, *PTV D2%* Near maximum dose of the PTV in EQD2, *PTV D50%* median dose of the PTV in EQD2, *PTV D98%* near minimum dose to the PTV in EQD2, *GTV D2%* near maximum dose to the gross tumor volume in EQD2, *IGRT* image-guided radiotherapy, *FB* free breathing, *iBH* internal breath-hold, *GTV* gross tumor volume, *PTV* planning target volume

The use of IGRT in FB vs in iBH demonstrated a strong association with the outcome (HR 6.3, 95% CI 1.39–28.55, p = 0.017). Neither the size of GTV (HR 1.00, 95% CI 0.99–1.00, p = 0.922) nor PTV (HR 1.00, 95% CI 0.99–1.00, p = 0.849) were significant predictors. Additionally, prior systemic therapy to SBRT showed no significant association with FFLP (HR 0.90, 95% CI 0.25–3.27, p = 0.872). Only SBRT to 11 lesions was administered with concurrent systemic therapies. Although the hazard ratio (HR) was extremely low (HR < 0.01), it was not statistically significant (p = 0.4).

After excluding variables associated with collinearity (supplementary Table 3), a multivariate Cox proportional hazards regression model was developed to assess the impact of PD, volume of GTV, and IGRT on the risk of events. The model demonstrated strong discriminatory ability, with a concordance index of 0.782, indicating robust predictive performance and log-rank test (p = 0.004).

Among the included variables, PD emerged as a significant predictor of outcomes, HR = 0.96 (95% CI 0.92–0.99, p = 0.026). In contrast, IGRT (FB vs iBH) showed a hazard ratio of 3.28 (95% CI 0.64–16.82, p = 0.155), indicating a potential, albeit statistically non-significant, association with increased risk. Finally, GTV volume was not significantly associated with the outcome (HR = 0.997, 95% CI 0.985–1.010, p = 0.677).

Three patients (3.9%) experienced Grade 3 toxicities following SBRT. The first patient developed Grade 3 cholestasis six weeks post-SBRT, necessitating biliary stenting. The second patient exhibited a greater than fivefold elevation in transaminases due to reactivation of immunotherapy-related hepatitis after SBRT and was managed with steroids. The third patient suffered post-SBRT necrosis of the metastasis complicated by superinfection; this occurred in the context of concurrent treatment with bevacizumab and fluoropyrimidines during SBRT. No Grade 4 or 5 toxicities were observed in the cohort.

### Modeling tumor control probability based on dose metrics

To evaluate the relationship between the various dose metrics to PTV and 1-year tumor control, we constructed four logistic regression models using PD, PTVmax, PTV D2%, and PTV D50% as predictors. The model fit and predictive performance were assessed using residual deviance and AIC.

**Fig. 3:**
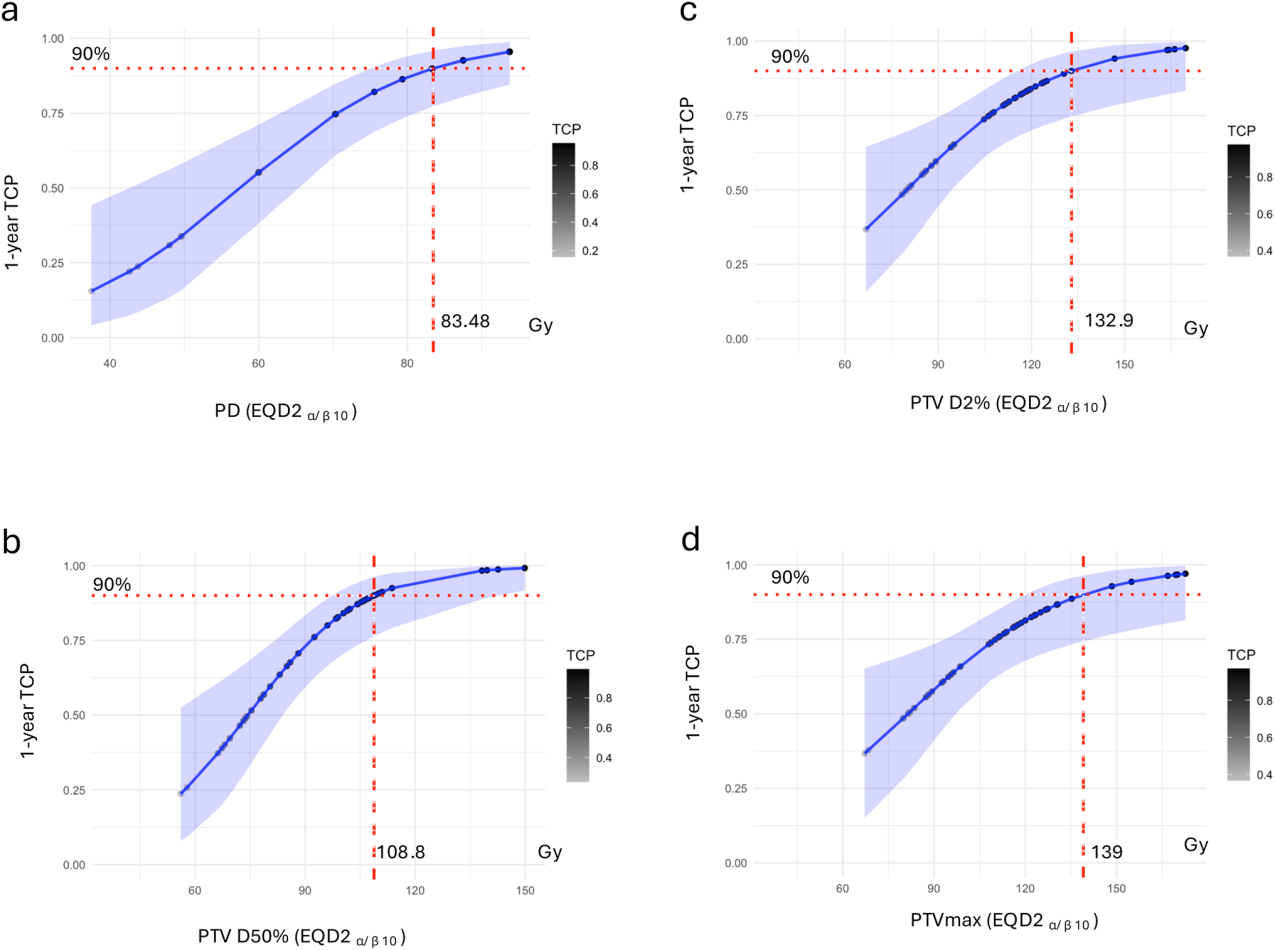
Logistic regression analysis of 1-year tumor control probability (TCP) as a function of various dose metrics, including PD (EQD2 α/β = 10), PTV D2% (EQD2 α/β = 10), PTV D50% (EQD2 α/β = 10), and PTVmax (EQD2 α/β = 10). Each plot displays the logistic regression curve (blue line) with shaded 95% confidence intervals (light blue area) and the predicted TCP values for individual data points (colored points). The dashed vertical red lines represent the dose corresponding to a 90% TCP, with the value annotated in gray. Horizontal dotted red lines indicate the TCP threshold of 90%

The PD model demonstrated the best fit to the data, with the lowest residual deviance (43.964) and AIC (47.964). PD was significantly associated with 1-year tumor control (coefficient = 0.085, p = 0.002), indicating that higher PD values increased the odds of tumor control. Among all models, the PD model provided the most robust predictive performance (Fig. 3; Table 3).

**Table 3:** Provides the results of statistical modeling, including coefficients (estimates), standard errors, p-values, residual deviance, and Akaike Information Criterion (AIC) for different dosimetric parameters

Model	Coefficient (estimate)	Std. error	p-value	Residual deviance	AIC
PD	0.085	0.028	0.002 **	43.964	47.964
PTVmax	0.038	0.017	0.024 *	50.245	54.245
PTVD2%	0.041	0.018	0.020 *	49.787	53.787
PTVD50%	0.064	0.022	0.004 **	45.623	49.623

*PD* prescribed dose as equivalent in 2 Gy fractions (α/β = 10) (EQD2), *PTVmax* maximum dose to the planning target volume as EQD2, *PTVD2%* near maximum dose to planning target volume as EQD2, *PTVD50%* median dose to planning target volume as EQD2, *AIC* Akaike Information Criterion

Significance levels: *p < 0.05, **p < 0.01

The model incorporating PTV D50% showed the second-best fit, with a residual deviance of 45.623 and AIC of 49.623. PTV D50% was also significantly associated with 1-year tumor control (coefficient = 0.064, p = 0.004), suggesting that higher D50% values positively influence tumor control, though the effect was slightly less pronounced than PD.

The PTV D2% and PTVmax models also demonstrated statistically significant associations with 1-year tumor control, with coefficients of 0.041 (p = 0.020) and 0.038 (p = 0.024), respectively. However, these models had higher residual deviances (49.787 and 50.245) and AIC values (53.787 and 54.245), indicating a slightly poorer fit than the PD and PTV D50% models.

Overall, the results highlight that PD to the periphery of PTV is the strongest and most reliable predictor of 1-year tumor control among the metrics evaluated.

A time-dependent ROC analysis was conducted to determine the optimal cutoff point for PD in predicting 1-year local control. The analysis identified an optimal threshold of 77.44 Gy, achieving a sensitivity of 92.3%. Kaplan–Meier survival analysis further revealed that patients treated with a PD ≤ 77.44 Gy had significantly worse FFLP rates compared to those receiving higher doses (p = 0.007), with the 1-year FFLP rate for patients with PD > 77.44 Gy was 96.8% (95% CI 90.8–100%), whereas patients with PD ≤ 77.44 Gy exhibited a substantially lower 1-year FFLP rate of 67.2% (95% CI 52.5–86.1%) (Fig. 2c).

## Discussion

This study provides a detailed analysis of SBRT for liver metastases in 76 patients with 101 lesions, highlighting key insights into survival outcomes, dosimetric parameters, prognostic factors, and modeling of tumor control probabilities. Our findings contribute to the growing body of evidence supporting SBRT as a highly effective and safe treatment modality for liver metastases.

The median OS in our study was 33 months, with 1-year and 3-year OS rates of 74.1% and 39.4%, respectively. These results are consistent with existing literature, which reports median OS following SBRT ranging from 16 to 33 months [25–30]. These results reflect not only the efficacy of SBRT in achieving local control but also advancements in systemic therapies and multidisciplinary management approaches [31]. As systemic treatments continue to evolve, the integration of SBRT provides an opportunity to further prolong survival and improve quality of life by achieving durable local control and potentially offering chemotherapy-free intervals [4, 5, 32, 33].

The durable FFLP rates observed in this cohort—86.4% at 6 months and 82.5% at 12 and 24 months—underscore the effectiveness of SBRT in maintaining local tumor control over time. Importantly, our univariate and multivariate analyses identified dosimetric parameters to PTV as significant predictors of FFLP. Among these, PD emerged as the most potent independent factor influencing tumor control, with an optimal threshold identified at 77.44 Gy as EQD2 _α/ β 10_ (a dose equivalent to the biologically effective dose “BED” 93 Gy). Patients receiving doses above this threshold demonstrated significantly higher 1-year FFLP rates (96.8%) than those receiving lower doses (67.2%). These findings align with prior studies emphasizing the importance of dose escalation in SBRT to overcome radioresistance and maximize tumor control [26, 28, 29, 34]. This strong correlation between dosimetric metrics and tumor control underscores the necessity for precise treatment planning with aggressive radiation dose to achieve optimal outcomes.

Our analysis revealed no significant relationship between FFLP and tumor volume metrics, such as GTV or PTV size, suggesting that tumor volume may play a less critical role than dosimetric factors in achieving local control. While this finding contrasts with earlier studies that identified tumor volume as a determinant of local control [28, 29], it likely reflects advancements in IGRT and the ability to deliver higher, more conformal radiation doses to larger tumors. This lack of correlation also underscores the versatility of SBRT in treating a wide range of lesion sizes, where other modalities, such as thermal ablation, often face limitations, particularly in managing larger or anatomically challenging tumors [35].

The comparison of FFLP between CRC and non-CRC metastases revealed no significant differences, challenging historical perceptions of CRC metastases as less responsive to radiation therapy [14, 36]. This finding is consistent with recent data demonstrating comparable outcomes across different primary tumor types when treated with SBRT [15]. These results underscore the potential of SBRT to provide effective local control agnostic to tumor origin, broadening its applicability in clinical practice.

Moreover, a significant strength of our study lies in incorporating advanced motion management techniques, particularly IGRT combined with iBH using a surface-guided RT (SGRT) system. Patients treated with IGRT-iBH demonstrated superior FFLP rates compared to those treated with FB IGRT, highlighting the critical role of motion management in minimizing tumor motion, improving target coverage, and sparing adjacent healthy tissues [17, 18, 20] and also supporting previous analysis, which showed the advanced motion management improved the local control [28]. These findings emphasize the importance of integrating advanced imaging and motion management strategies to optimize the therapeutic ratio of SBRT.

Beyond survival and tumor control outcomes, our study explored the modeling of tumor control probabilities as a function of dosimetric parameters. Logistic regression models revealed that PD was the most robust predictor of 1-year tumor control, with the lowest residual deviance and AIC values. Models incorporating PTV D50%, PTVmax, and PTV D2% also demonstrated statistically significant associations with tumor control but were less predictive than the PD model. Our models align with the previous modeling efforts by Ohri et al., who reported a 90% 2-year TCP with a Dmax of 180 Gy as BED, equivalent to 150 Gy when converted to EQD2 _α/β 10_, in comparison, our 1-year TCP was 139 Gy as EQD2 _α/β = 10_ [34].

These results highlight the importance of precise dosimetric planning and advanced imaging strategies in optimizing treatment outcomes for patients undergoing SBRT.

The safety profile observed in our study further supports the utility of SBRT in managing liver metastases. Grade 3 toxicities were observed in only 3.9% of patients, with no Grade 4 or 5 events, consistent with previous reports highlighting the tolerability of SBRT [13]. Even at high dose levels, the low toxicity rates reflect advancements in treatment delivery techniques, including IGRT and motion management. These allow for precise targeting and sparing of normal tissues with caution when combining anti-angiogenic therapies during SBRT.

### Limitations of the study

Despite the promising results, our study is limited by its retrospective nature and relatively short median follow-up period. Long-term outcomes and potential late toxicities warrant further investigation. Additionally, the heterogeneity of primary tumor types and prior treatments in our cohort may influence the generalizability of our findings.

## Conclusion

In conclusion, our study highlights the efficacy, safety, and dosimetric determinants of SBRT in the management of liver metastases. The findings reinforce the critical role of advanced imaging and motion management in optimizing outcomes and underscore the importance of dosimetric modeling in personalizing treatment. Future research should focus on refining dose–response relationships and validating predictive models. The evolving landscape of metastatic cancer management provides an opportunity to expand the role of SBRT as a cornerstone of precision oncology.

## Supplementary Information

Below is the link to the electronic supplementary material.

Supplementary file1 (DOCX 20 kb)

## Data Availability

Data used in the analysis is available upon request from the corresponding author.
