# Supplementary material for: Enhancing tumor control in liver metastases treated with SBRT: dosimetric predictors and clinical outcomes from a single-center analysis
Source: Clin Exp Metastasis. 2025 Apr 26;42(3):28. doi: 10.1007/s10585-025-10344-3 (PMC12033208; doi:10.1007/s10585-025-10344-3)
Supplement: Supplementary file 1 — Supplementary file1 (DOCX 20 kb) [file 10585_2025_10344_MOESM1_ESM.docx]

| Organ at Risk | Parameter | 3 Fractions in Gy | 5 Fractions in Gy | 8 Fractions in Gy | 12 Fractions  In Gy |
| --- | --- | --- | --- | --- | --- |
| Stomach/Duodenum /Jejunum / Ileum | D 0.1 cm^3^ | 30 | 32 | 38.4 | 45 |
|  | D 5cm cm^3^ | 22.5 | 31.5 | 38 | 44.4 |
| Colon | D 1 cm^3^ | 40 | 50 | 60 | 66 |
| Liver | D_mean_  Ciritiacla cvolume (700 cm^3^) | ≤ 15  ≤ 15 | < 20 (preferred ≤ 15)  ≤ 21 | < 22 (preferred ≤ 18)  ≤ 24 | < 24  ≤ 26 |
| Common bile duct | D 0.1 cm^3^ | 35 | 40 | 42.2 | 49.8 |

Supplementally, table 1 dose constraints to organs at risk

Supplementally, table 2 Doses and Fractionations per lesion

| Fractionation schedules | Prescribed pyhsical dose | EQD2 / BED 10 | Results oft he planning |
| --- | --- | --- | --- |
| 29 lesions planned for 3x 12.5-15 Gy | 37.5-45 Gy | 70.31-93.75 Gy/ 84.38-112.5 Gy | - 26 cases met constraints for OAR  - 3 cases reduced to 3 x 10 :30 Gy(EQD2: 50 Gy, BED 10: 60 Gy) to meet the constrains for OAR |
| 50 lesions planned for 5x 8-10 | 40-50 Gy | 60-83.3 Gy /  72-100 Gy | - 43 cases met the constraints for OAR  - 7 cases were reduced to 5x 6-7.5 : 30 -37.5 Gy (EQD2: 40-54.69 Gy, BED 48: 65.63 Gy) to meet the constrains for OAR |
| 16 lesions planned for 8x 6 -7.5 | 48-60 Gy | 64-87.5 Gy/  76.8-105 Gy | 15 cases met the constraints for OAR  1 cases were reduced to 8x 4.5: 36 Gy (EQD2: 43.5 Gy, BED 10 52.2 Gy to meet the constrains for OAR |
| 6 lesions were planned for 12x 4-5.5 | 48-66 | 56 – 85.25 Gy/  67.2 – 102.3 Gy | All lesions were met the constrains for OAR |

|  | **PDEQD2** | **PTVmaxEQD2** | **PTVd2EQD2** | **PTV50EQD2** | **PTV95EQD2** | **PTV98EQD2** | **GTV2DEQD2** | **IGRT** | **GTV in mL** | **PTV in mL** | **systemic therapy** | **Tumor_other** |
| --- | --- | --- | --- | --- | --- | --- | --- | --- | --- | --- | --- | --- |
| **PDEQD2** | 1.0 | 0.8016870923958701 | 0.8222038303823663 | 0.910008062106264 | 0.8310040660253346 | 0.7611343956614837 | 0.8181946443769298 | -0.3876225196520074 | 0.06805258299778497 | 0.06737980607481255 | 0.2632832909754254 | -0.3174548935464265 |
| **PTVmaxEQD2** | 0.8016870923958701 | 1.0 | 0.9952805830957343 | 0.93594436777754 | 0.7721749093495701 | 0.7055148148234103 | 0.9932405897215255 | -0.11725686132497848 | 0.03625235511498135 | -0.023853028263389173 | 0.2042823350533526 | -0.20511258357096046 |
| **PTVd2EQD2** | 0.8222038303823663 | 0.9952805830957343 | 1.0 | 0.9526605804773793 | 0.7885831609722269 | 0.7209310667350805 | 0.9973841379266394 | -0.13365202245856642 | 0.03585609295873929 | -0.017908644476946317 | 0.212776190981988 | -0.2237284665492179 |
| **PTV50EQD2** | 0.910008062106264 | 0.93594436777754 | 0.9526605804773793 | 1.0 | 0.863845278756552 | 0.7852509480825921 | 0.9490689541698146 | -0.23451958937184178 | 0.06507025729480778 | 0.012508329765201309 | 0.22999752768431064 | -0.310269271079152 |
| **PTV95EQD2** | 0.8310040660253346 | 0.7721749093495701 | 0.7885831609722269 | 0.863845278756552 | 1.0 | 0.9797483272571005 | 0.7782252828169364 | -0.15049101592241285 | -0.03934379708648289 | 0.068774809328202 | 0.2131834627440105 | -0.3014757909101757 |
| **PTV98EQD2** | 0.7611343956614837 | 0.7055148148234103 | 0.7209310667350805 | 0.7852509480825921 | 0.9797483272571005 | 1.0 | 0.7135994620222335 | -0.10597952724680451 | -0.1024630841915678 | 0.0783115290148381 | 0.20395722567762395 | -0.26484374490436846 |
| **GTV2DEQD2** | 0.8181946443769298 | 0.9932405897215255 | 0.9973841379266394 | 0.9490689541698146 | 0.7782252828169364 | 0.7135994620222335 | 1.0 | -0.12621740782668348 | 0.03768031752898595 | -0.015550287085473445 | 0.21339653097974304 | -0.22733943057734307 |
| **IGRT** | -0.3876225196520074 | -0.11725686132497848 | -0.13365202245856642 | -0.23451958937184178 | -0.15049101592241285 | -0.10597952724680451 | -0.12621740782668348 | 1.0 | -0.1020453537539836 | -0.1010331734859727 | -0.17825602397684537 | 0.08043217286914764 |
| **GTV in mL** | 0.06805258299778497 | 0.03625235511498135 | 0.03585609295873929 | 0.06507025729480778 | -0.03934379708648289 | -0.1024630841915678 | 0.03768031752898595 | -0.1020453537539836 | 1.0 | -0.010380977910137843 | 0.053518554270298904 | -0.09689278070878438 |
| **PTV in mL** | 0.06737980607481255 | -0.023853028263389173 | -0.017908644476946317 | 0.012508329765201309 | 0.068774809328202 | 0.0783115290148381 | -0.015550287085473445 | -0.1010331734859727 | -0.010380977910137843 | 1.0 | 0.05366943437808464 | 0.10359376613220328 |
| **systemic therapy** | 0.2632832909754254 | 0.2042823350533526 | 0.212776190981988 | 0.22999752768431064 | 0.2131834627440105 | 0.20395722567762395 | 0.21339653097974304 | -0.17825602397684537 | 0.053518554270298904 | 0.05366943437808464 | 1.0 | -0.010623825068408695 |
| **Tumor_other** | -0.3174548935464265 | -0.20511258357096046 | -0.2237284665492179 | -0.310269271079152 | -0.3014757909101757 | -0.26484374490436846 | -0.22733943057734307 | 0.08043217286914764 | -0.09689278070878438 | 0.10359376613220328 | -0.010623825068408695 | 1.0 |

Supplementally, table 3 shows the correlation coefficient for the variables
